# Association between vitamin D status and asthma control levels among children and adolescents: a retrospective cross sectional study in Saudi Arabia

**DOI:** 10.1186/s12887-025-05969-y

**Published:** 2025-08-01

**Authors:** Abdullah Alolayan, Osama Al-Wutayd

**Affiliations:** 1 Department of Pediatrics, College of Medicine, Qassim University, Buraydah, Saudi Arabia; 2 Department of Family and Community Medicine, College of Medicine, Qassim University, Buraydah, Saudi Arabia

**Keywords:** Adolescent, Asthma control, BMI, Children, Seasonal variation, Retrospective study, Vitamin D level

## Abstract

**Background:**

Asthma in children is one of the most common chronic disorders, with a significant impact on patients and their families. Few studies have been conducted in Saudi Arabia. This study aimed to determine the association between vitamin D status and asthma control. Additionally, it investigates the level of 25-hydroxy vitamin D (25(OH)D) and its associated factors among asthmatic children and adolescents in Qassim region.

**Methods:**

This retrospective cross sectional study identified patients aged 1–15 years from January 2018 to January 2024, who attended pediatric clinics at Sulaiman Alhabib Hospital in Buraydah city, Qassim region, Saudi Arabia. Data on age, gender, body mass index (BMI), 25(OH)D levels, and seasonal variations were obtained from hospital records.

**Results:**

A total of 431 asthmatic patients were identified, with a median [IQR] age of 9 [7–12] years. Among them, 268 (62.2%) had vitamin D deficiency (25(OH)D < 50 nmol/L; <20 ng/mL), with an overall median [IQR] 25(OH)D level of 42.6 nmol/L [31.6–58.8]; 17.0 ng/mL [12.6–23.5]. The median serum 25(OH)D level was highest in autumn and lowest in spring. Multiple linear regression analysis showed that participants aged 1–7 years (β = 19.3, 95% CI: 14.4 to 24.1, *p* < 0.001) and male participants (β = 4.9, 95% CI: 0.4 to 9.4, *p* = 0.033) had significantly higher 25(OH)D levels, whereas obese patients (β = -8.6, 95% CI: -14.7 to -2.6, *p* = 0.005), and those with 25(OH)D levels measured during the spring season (β = -6.5, 95% CI: -12.4 to -0.7, *p* = 0.029) had significantly lower 25(OH)D levels. The median [IQR] serum 25(OH)D levels for controlled, partially controlled, and uncontrolled asthma were 41.8 nmol/L [31–58]; 16.7 ng/mL [12.4–23.2], 43.6 nmol/L [30–61]; 17.4 ng/mL [12-24.4], and 45.2 nmol/L [30–57]; 18.1 ng/mL [12-22.8], respectively. Age, BMI, 25(OH)D levels, and seasonal variations were not found to be associated with asthma control levels.

**Conclusion:**

Vitamin D deficiency was common among asthmatic children and adolescents. Lack of statistical association was observed between independent variables (age, gender, 25(OH)D levels, seasonal variations, and BMI) and asthma control in this setting. Routine assessment of vitamin D levels may not predict asthma control status.

## Introduction

Asthma in children is one of the most common disorders that is characterized by reversible airway obstruction resulting from the interaction of genetic and environmental factors [1]. The prevalence of asthma is increasing globally [2] and in Saudi Arabia [3], with regional variations among Saudi children ranging from 9 to 33.7% [4]. This condition has a significant impact on patients and their families, leading to absenteeism from school and work, reduced quality of life, frequent hospitalizations, emergency room visits, and, in severe cases, deaths [5]. Asthma is often triggered by exposure to environmental antigens, causing inappropriate immune reactions characterized by wheezing and respiratory distress [6]. By controlling both innate and adaptive immune responses, vitamin D plays an immunomodulatory role in the pathophysiology of asthma. It reduces eosinophilic inflammation and airway hyperresponsiveness by binding to the vitamin D receptor (VDR) expressed on immune cells, which in turn stimulates the differentiation of regulatory T cells and inhibits pro-inflammatory Th2 and Th17 responses [7, 8]. Moreover, vitamin D promotes the synthesis of antimicrobial peptides, which may minimize respiratory infections that lead to flare-ups of asthma. Furthermore, it upregulates IL-10, an anti-inflammatory cytokine that reduces bronchial inflammation, while downregulating important inflammatory cytokines like IL-4, IL-5, and IL-13 [9–12]. Despite the abundant sunshine in Saudi Arabia, vitamin D deficiency is common due to limited sun exposure [13], and study in western region of Saudi Arabia reported an association between vitamin D deficiency and asthma control [14], while other studies in Western, Southwest, and Riyadh regions did not find an association [15–17]. Up to our knowledge, there is no data from Qassim region, limited adolescent inclusion, and lack of seasonal analysis, therefore, this study aimed to determine the association between vitamin D status and asthma control, as well as examining the 25-hydroxy vitamin D (25(OH)D) levels and their associated factors among Saudi asthmatic children and adolescents in Qassim region.

## Methods

### Study design and setting

This study was a retrospective cross sectional study conducted at the pediatric clinics of Sulaiman Alhabib Hospital in the Qassim region from 2018 to 2024. Sulaiman Alhabib Hospital is the largest private hospital in the Qassim region, with a capacity of 173 beds [18]. The hospital is located in Buraydah, the capital and largest city of the Qassim region, with a population of approximately 1.3 million [19]. The Qassim region is one of the 13 administrative regions of Saud Arabia and is known for its agricultural significance. It features a desert climate with low humidity and frequent sandstorms. The region is centrally located in the country, approximately 350 km from Riyadh, the capital city of Saudi Arabia [20].

### Subject recruitment

**Fig. 1:**
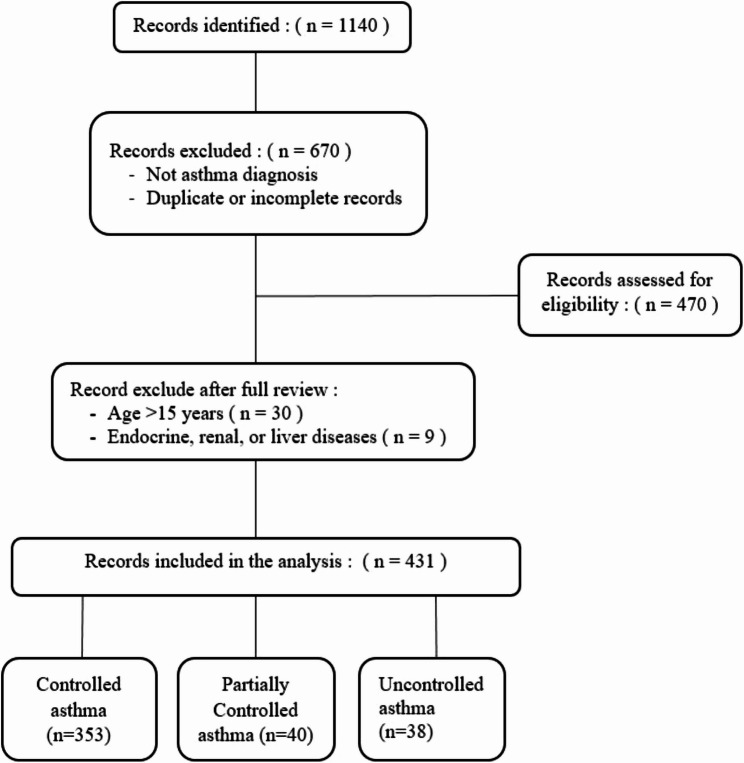
Flow chart of patient selection

Asthma was identified based on the ICD-10 diagnostic code: J45. Alternatively, clinical criteria that mentioned by the global initiative for asthma (GINA) 2024 guidelines including a history of characteristics respiratory symptoms such as wheeze, shortness of breath, chest tightness, and cough [21]. Medical records of children and adolescents aged 1–15 years who were diagnosed with asthma from January 2018 to January 2024 were reviewed if serum 25(OH)D levels were available. Patients with other conditions that might affect 25(OH)D levels, such as endocrine, renal, bone or liver diseases were excluded. Data on age, gender, height, weight, and 25(OH)D levels were obtained from hospital records by nurses and subsequently verified by a pediatric pulmonology consultant (Fig. 1).

### Definitions and measurements

Patients with asthma were classified as having controlled, partially controlled, and uncontrolled asthma based on the GINA classification, updated May 2024 version [22]. The measurement of 25(OH)D levels occurred during follow up and cutoff points were as follows: a plasma 25(OH)D level of < 50 nmol/L (< 20 ng/mL) was categorized as deficient; 50–74 nmol/L (20–29 ng/mL), insufficient; and ≥ 75 nmol/L (≥ 30 ng/mL), adequate [23]. BMI was categorized as underweight (< 5th percentile for age); normal weight (5th to 85th percentile); overweight (85th to 95th percentile); and obese (> 95th percentile) according to the Saudi growth chart [24]. The 25(OH)D level was measured using enzyme-linked immunosorbent assay (ELISA) kits (Euroimmun, Lübeck, Germany) according to the manufacturer’s instructions. Six calibrators, with concentrations ranging from 0 to 120 ng/mL, were used, and the manufacturer-recommended quality control measures were strictly followed. Seasonal variation was defined based on the season during which the 25(OH)D levels were measured and was categorized from June to August (summer); from September to November (autumn); from December to February (winter); and from March to May (spring).

### Sample size calculation

The sample size was calculated using one-way analysis of variance in Stata software (version 17), guided by the reported mean 25(OH)D levels of 53, 33, and 19 nmol/L for the controlled, partially controlled, and uncontrolled asthma in Saudi Arabia, respectively [14]. We assumed that the significance level was 0.05, and desired power at 0.80 with a ratio of approximately 5:1:1 across asthma groups to maximize the statistical power and compensate for the few cases in groups of partially controlled and uncontrolled asthma. The minimum sample size of 266 was needed (N1 = 190, N2 = 38, and N3 = 38).

### Statistical analysis

Statistical analysis was performed using Stata software (version 17). The Shapiro–Wilk test was conducted to determine the distribution of continuous variables. Numbers and percentages (%) were presented for categorical data. Mean and standard deviation (SD) were presented for normally distributed data. Median and interquartile range [IQR] were presented for non-normally distributed data. Simple linear regression analysis was performed to assess serum 25(OH)D levels as the dependent continuous variable, with demographic and clinical characteristics as independent variables. Variables with a p-value of ≤ 0.20 (age group, gender, BMI, and seasons) were constructed in the multiple linear regression model. Beta coefficient (β) with 95% confidence intervals (CIs) was reported. Furthermore, subjects were divided into three groups: controlled, partially controlled, and uncontrolled asthma. The Kruskal–Wallis test was used for non-normally distributed continuous data, and the chi-square test was used for categorical data. A p-value of ≤ 0.05 was considered to indicate strong evidence against the null hypothesis.

## Results

**Fig. 2:**
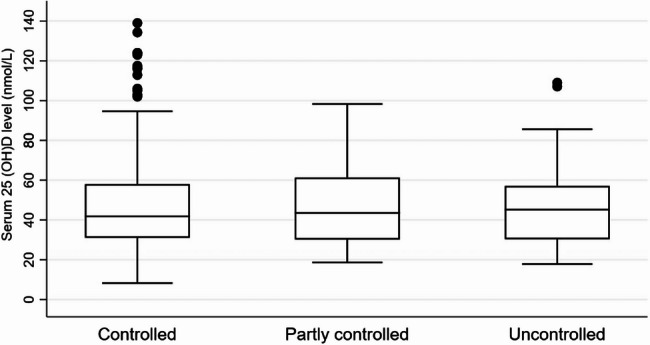
This box plot showed the median [IQR] of serum 25(OH)D levels (nmol/L) in controlled, partially controlled, and uncontrolled asthma (*p* = 0.764); The Kruskal–Wallis test

**Fig. 3:**
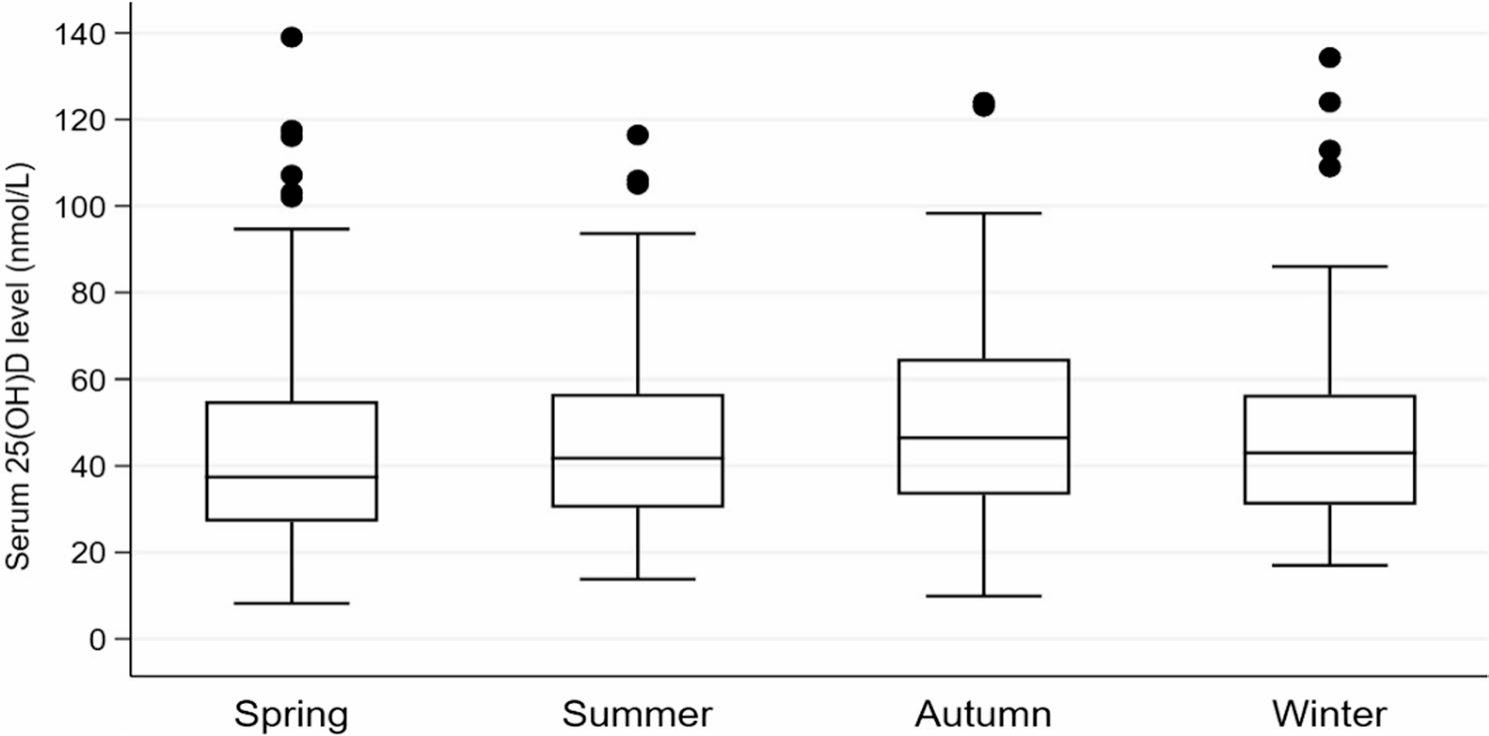
This box plot showed the median [IQR] of serum 25(OH)D levels (nmol/L) of asthmatic children and adolescents in spring, summer, autumn, and winter season

A total of 431 asthmatic children and adolescents aged 1–15 years were identified in this study. The median [IQR] age of the participants was 9 [7–12] years; 245 (56.8%) were males. Among the participants, 59 (13.7%) were overweight and 73 (16.9%) were obese. The median [IQR] serum 25(OH)D level was 42.6 nmol/L [31.6–58.8]; 17.0 ng/mL [12.6–23.5], and 268 (62.2%) had vitamin D deficiency (25(OH)D < 50 nmol/L; <20 ng/mL). According to the level of asthma control, 353 (81.9%) had controlled asthma, 40 (9.3%) had partially controlled asthma, and 38 (8.8%) had uncontrolled asthma (Table 1). The median [IQR] serum 25(OH)D levels were 41.8 nmol/L [31–58]; 16.7 ng/mL [12.4–23.2] for controlled asthma, 43.6 nmol/L [30–61]; 17.4 ng/mL [12-24.2] for partially controlled asthma, and 45.2 nmol/L [30–57]; 18.1 ng/mL [12-22.8] for uncontrolled asthma (*p* = 0.764) (Fig. 2). A comparison of the controlled, partially controlled, and uncontrolled asthma groups showed no statistical difference in age, gender, BMI, or 25(OH)D levels (Table 2). Regarding associated factors with 25(OH)D levels, simple linear regression analysis indicated that the age group 1–7 years (β = 19.6, 95% CI: 14.8 to 24.4, *p* < 0.001) had significantly higher 25(OH)D levels, while obese participants (β = −11.9, 95% CI: −18.3 to −5.5, *p* < 0.001) had significantly lower 25(OH)D levels. Multiple linear regression analysis further demonstrated that participants aged 1–7 years (β = 19.3, 95% CI: 14.4 to 24.1, *p* < 0.001) and male participants (β = 4.9, 95% CI: 0.4 to 9.4, *p* = 0.033) had significantly higher 25(OH)D levels, whereas obese participants (β = −8.6, 95% CI: −14.7 to −2.6, *p* = 0.005) had significantly lower 25(OH)D levels. Likewise, 25(OH)D levels were significantly lower at spring season (β = −6.1, 95% CI: −12.4 to −0.7, *p* = 0.029) compared to autumn (Table 3). According to seasonal variation, the median [IQR] serum 25(OH)D level was 37.4 nmol/L [27–55]; 14.9 ng/mL [10.8–22] in spring, 41.8 nmol/L [30–57]; 16.7 ng/mL [12-22.8] in summer, 46.5 nmol/L [33–65]; 18.6 ng/mL [13.2–26] in autumn, and 43 nmol/L [31–56]; 17.2 ng/mL [12.4–22.4] in winter (Fig. 3).

**Table 1:** Demographic and clinical characteristics of the asthmatic children and adolescents (*n* = 431)

Characteristics		Median	IQR
Age		9	7–12
Weight		28.4	19–48
Height		131.8	112–149
25(OH)D level (nmol/L)		42.6	31.6–58.8

		**Number**	**%**
Gender	Male	245	56.8
	Female	186	43.2
BMI	Normal	299	69.4
	Overweight	59	13.7
	Obese	73	16.9
Vitamin D status	Deficiency (< 50 nmol/L)	268	62.2
	Insufficiency (50–74 nmol/L)	115	26.7
	Sufficiency (≥ 75 nmol/L)	48	11.1
Asthma control levels	Controlled	353	81.9
	Partially controlled	40	9.3
	Uncontrolled	38	8.8
Seasons	Spring	113	26.2
	Summer	81	18.8
	Autumn	133	30.9
	Winter	104	24.1

**Table 2:** Comparison of demographic and clinical characteristics according to asthma control levels

Characteristics		Controlled (*n* = 353)	Partially controlled (*n* = 40)	Uncontrolled (*n* = 38)	*P*-value
	Median [IQR] values were compared using Kruskal–Wallis test				
Age		9 [7–13]	9.5 [7–12.5]	8 [6–11]	0.362
25(OH)D level nmol/L		41.8 [31–58]	43.6 [30–61]	45.2 [30–57]	0.764
	Number (%) values were compared using chi-square test				
Gender	Male	195 (79.6)	28 (11.4)	22 (9)	0.201
	Female	158 (84.9)	12 (6.5)	16 (8.6)	
BMI	Normal	251 (84)	24 (8)	24 (8)	0.295
	Overweight	47 (79.7)	8 (13.5)	4 (6.8)	
	Obese	55 (75.3)	8 (11)	10 (13.7)	
Vitamin D status	Deficiency (< 50 nmol/L)	218 (81.3)	26 (9.7)	24 (9)	0.354
	Insufficiency (50–74 nmol/L)	96 (83.5)	12 (10.4)	7 (6.1)	
	Sufficiency (≥ 75 nmol/L)	39 (81.3)	2 (4.2)	7 (14.6)	
Season	Spring	94 (83.2)	12 (10.6)	7 (6.2)	0.931
	Summer	66 (81.5)	8 (9.9)	7 (8.6)	
	Autumn	109 (81.9)	11 (8.3)	13 (9.8)	
	Winter	84 (80.8)	9 (8.7)	11 (10.6)	

**Table 3:** Simple and multiple linear regression analysis of serum 25(OH)D levels with demographic and clinical characteristics among asthmatic children and adolescents

Variables		Median (nmol/L) [IQR]	Simple linear regression			Multiple linear regression		
			**β**	**95% CI**	***P*** value	**β**	**95% CI**	***P*** value
Age group	1–7 years	54.8 [42–73]	19.6	14.8,24.4	< 0.001	19.3	14.4,24.1	< 0.001
	8–15 years	36.7 [28–51]	reference					
Gender	Male	45 [34–60]	3.3	−1.6,8.1	0.185	4.9	0.4,9.4	0.033
	Female	38.1 [28–56]	reference					
BMI	Normal	45 [33–62]	reference					
	Overweight	43 [32–60]	−3.1	−10.1,3.9	0.388	−1.3	−7.8,5.3	0.700
	Obesity	34 [27–44]	−11.9	−18.3,−5.5	< 0.001	−8.6	−14.7,−2.6	0.005
Seasons	Spring	37.4 [27–55]	−6.1	−12.4,0.3	0.061	−6.5	−12.4,−0.7	0.029
	Summer	41.8 [30–57]	−1.8	−8.8,5.2	0.622	−4.2	−10.7,2.2	0.199
	Autumn	46.5 [33–65]	reference					
	Winter	43 [31–57]	−3.2	−9.7,3.3	0.337	−4.2	−10.2,1.8	0.174

## Discussion

The main finding of the present study revealed that the median 25(OH)D level showed small absolute differences across asthma control levels, with 1.8 nmol/L between controlled and partially controlled asthma, 3.4 nmol/L between controlled and uncontrolled asthma, and 1.6 nmol/L between partially and uncontrolled asthma; this difference was not statistically significant. This finding aligns with several studies conducted in different regions of Saudi Arabia [15–17], while another study found an association between low vitamin D levels and poor asthma control [14]. Variations in the literature may be due to differences in the definition of asthma control, small sample sizes, single vitamin D measurements, and seasonal variations. However, the present study consistent with a meta-analysis that include 14 RCTs which found no effect of vitamin D supplementation on asthma control [25]. In this study, we did not observe a statistically significant association between vitamin D levels and asthma control. Overweight and obesity have been negatively associated with asthma control in various studies; however, this association is complex, and some studies have found no effect of overweight/obesity on asthma control. In our study, the prevalence of obesity was higher in the controlled asthma group compared to the partially controlled and uncontrolled asthma groups. However, we did not find an association between overweight/obesity and asthma control, possibly due to the relatively small number of obese subjects in our study. This finding is consistent with studies conducted in Saudi Arabia [14, 15, 26]. Furthermore, a meta-analysis including 46,070 asthmatic children and adolescents showed no association between overweight/obesity and asthma control [27]. Whereas, other studies from Saudi Arabia [28] and neighboring country of United Arab Emirates reported an association [29]. Contradictory findings may be explained by genetic and environmental factors, such as race/ethnicity differences in access to clinics and emergency departments for asthmatic children [30]. Additionally, some studies used self-reported weight and height, whereas our study measured these in a clinical setting. We found vitamin D deficiency is common among children and adolescent with asthma in Saudi Arabia. The majority of participants had vitamin D deficiency, followed by approximately one-quarter with vitamin D insufficiency and only a few participants with vitamin D sufficiency. This finding is in the line with studies conducted in Saudi Arabia and the neighboring country of Qatar among asthmatic patients [15, 31]. Young children and boys had higher vitamin D levels, consistent with studies showing that vitamin D levels decrease with age and reach their lowest levels in adolescents [32, 33]. Boys had higher vitamin D levels than girls, likely due to greater engagement in outdoor activities [32–36]. Obese subjects had lower vitamin D levels compared to those with normal BMI, which aligns with various studies [37, 38]. It has been documented that vitamin D is more likely to be sequestered in adipose tissue rather than circulating in the bloodstream, explaining its deficiency in obese subjects [39]. Finally, vitamin D levels were found to be deficient during the spring season. The reason for low vitamin D levels in the spring season is not clear but could be attributed to environmental and biological factors. In Saudi Arabia, the spring season is characterized by high ozone content, which interferes with UV-B rays [40]. Also, spring follows a rainy winter season, which is often cloudy. These clouds, filled with water droplets, can interfere with UV-B rays and reflect most of them back into space [41]. Consequently, less UV-B is available for vitamin D synthesis during the winter season, which also features more outdoor activity. Regardless of the season, the duration and timing of sun exposure can affect the rate of vitamin D synthesis [42–44]. Furthermore, skin type and melanin content can affect the penetration of UV-B into the skin and its availability for vitamin D synthesis [45]. Dietary factors and lifestyle also play a role [46]. To the best of our knowledge, this is the largest study conducted in Saudi Arabia among children and adolescents with asthma, and data were obtained from clinical reports rather than self-reported diagnoses, weight, or height. Furthermore, it is the only study in Saudi Arabia that considered seasonal variation among asthmatic patients. However, there are some limitations. This is a retrospective, single-center, cross sectional design which limited the generalizability of study and reverse causation cannot be excluded, and factors such as viral infections, genetic factors, and skin color were not evaluated. Furthermore, socioeconomic status, inhaled steroid dose, sun exposure habits, dietary intake, outdoor activity, and vitamin D supplementation were not assessed. A prospective study is recommended to establish a temporal relationship and measure 25(OH)D levels before starting treatment.

## Conclusion

Vitamin D deficiency was common among asthmatic children and adolescents. Male and young participants were positively associated with 25(OH)D levels, whereas obese participants and spring season were negatively associated. The median 25(OH)D level showed small absolute differences across asthma control levels and no statistically significant association was observed between independent variables (age, gender, 25(OH)D levels, seasonal variations, and BMI) and asthma control in this setting. Thus, routine assessment of vitamin D levels may not predict asthma control status.

## Data Availability

No datasets were generated or analysed during the current study.
